# Variation in cycling exercise mechanical efficiency in the HERITAGE Family Study

**DOI:** 10.14814/phy2.70626

**Published:** 2025-10-29

**Authors:** Angelo Tremblay, Claude Bouchard, Mark A. Sarzynski, James S. Skinner, Élisa Marin‐Couture, Patrick Schrauwen, Denis R. Joanisse, Guy Thibault, Marie‐Eve Mathieu, Louis Pérusse

**Affiliations:** ^1^ Department of Kinesiology, Faculty of Medicine Laval University Quebec Quebec Canada; ^2^ Centre Nutrition, Santé et Société (NUTRISS), Institute of Nutrition and Functional Foods (INAF) Laval University Quebec Quebec Canada; ^3^ Pennington Biomedical Research Center Baton Rouge Louisiana USA; ^4^ Department of Exercise Science, Arnold School of Public Health University of South Carolina Columbia South Carolina USA; ^5^ Department of Kinesiology Indiana University Bloomington Indiana USA; ^6^ Institute for Clinical Diabetology, German Diabetes Center Leibniz Institute for Diabetes Research at Heinrich Heine University Düsseldorf Düsseldorf Germany; ^7^ School of Kinesiology and Physical Activity Sciences University of Montreal Quebec Quebec Canada

**Keywords:** body composition, cycling mechanical efficiency, exercise training, glucose, sex

## Abstract

The objectives of the study were to assess the effects of cycling endurance exercise training and sex on cycling exercise mechanical efficiency (ME) and to explore its associations with body composition, glycemic profile, and skeletal muscle characteristics. Subjects were males (*n* = 371) and females (*n* = 465) of European (ED) and African descent (AD) of the HERITAGE Family Study. The measurements were repeated after a 20‐week controlled endurance exercise program. A substudy involving 78 participants from 19 ED families was used to evaluate associations between skeletal muscle traits and variations in ME. An increase in ME of 4%–5% was induced by training and was comparable between sexes. Higher values of ME were associated with reduced body weight and fatness and fasting plasma glucose and insulin, both before and following endurance training, independently of cardiorespiratory fitness. There was no association between muscle fiber type or capillarity and ME. There was also no consistent muscle glycolysis and oxidative enzymatic profile associated with ME. The results of this study show that cycling exercise ME is trainable in nonathletes. ME variations in the sedentary state and in response to an exercise training program are associated with favorable body composition and glycemic profiles. Further research is needed to better understand its biological determinants.

## 1 INTRODUCTION

Mechanical efficiency (ME) measured during cycling exercise is calculated from the amount of external work relative to oxygen consumption (VO_2_). Its variations are influenced by both biomechanical and biological factors. Its most obvious determinant is the ability to cycle on a stationary bike since improved skills of execution allow for an increase in work output for a given VO_2_.

Exercise‐training is also known to influence ME. In this regard, high‐intensity training increased gross efficiency in competitive male cyclists (Hopker et al., 2010). Muscle efficiency improves over time in world‐class cyclists (Santalla et al., 2009), whereas favorable changes are noted in cycling efficiency throughout a competitive season (Hopker et al., 2009). Similarly, high‐intensity intermittent training improved ME in both young and older adults, suggesting its effectiveness across age groups (Jabbour et al., 2017). Despite the consistency of observations, there are gaps in the literature as studies have focused mainly on trained or elite cyclists, with limited research on sedentary or recreationally active people. Therefore, a first objective of this study was to assess the effects of an endurance training program performed on stationary bikes on ME in sedentary male and female adults.

Little is known on the correlates and biomarkers of human ME variation. These include mitochondrial efficiency, which is determined by the coupling between oxidative phosphorylation and ATP production. Mitochondrial uncoupling proteins, notably UCP2 and UCP3 in muscle, have been suggested to decrease energy efficiency (Boss et al., 1997; Hesselink et al., 2002). Additionally, diet composition could play a role in ME variations via its impact on the composition of the substrate mix oxidized since the ATP formed/VO_2_ ratio is slightly greater for carbohydrate than for lipid oxidation. In clinical studies, ME was investigated for its potential associations with body composition and metabolic indicators. In children with obesity, significant negative associations between ME and plasma low‐density lipoprotein cholesterol and glucose concentrations were observed (Franssen et al., 2021). This is concordant with the finding that the increase in ME induced by exercise training is associated with increases in insulin sensitivity and cardiorespiratory fitness (Jabbour & Iancu, 2015). Recently, we evaluated the relationship between cycling ME, body composition, and glycemic profile in participants of the Quebec Family Study (Marin‐Couture et al., 2024). We confirmed the previous findings but noted that differences in body composition and glycemic profile between tertiles of ME remained statistically significant after adjustments for current physical activity level and cardiorespiratory fitness. This finding suggests that the potential influence of ME on metabolic indicators is partly independent of habitual physical activity level and fitness. Thus, the present study aims also to investigate the associations between cycling ME and body composition or glycemic profile in participants at baseline (when subjects were sedentary) and then after 20 weeks of endurance training during which they became much more familiar with cycling.

Research suggests that variations in ME are associated with skeletal muscle fiber types and marker enzymes of energy metabolism. A high proportion of type 1 fibers was found to be associated with a higher exercise ME (Horowitz et al., 1994; Layec et al., 2011; Pleguezuelos et al., 2021; Umberger et al., 2006), which may be attributable to mitochondrial content (Hesselink & Schrauwen, 2005; Schrauwen et al., 1999). In a weight‐reducing program, the decrease in the energy cost of cycle exercise exceeded the decrease in ATP flux, reflecting an increase in muscle work efficiency (Rosenbaum et al., 2003). In a subsequent study, an increase in skeletal muscle ME was related to significant declines in glycolytic enzyme activities, especially the ratio of muscle phosphofructokinase/cytochrome c oxidase activity (Goldsmith et al., 2010). Thus, skeletal muscle fiber type composition and metabolism may influence ME under certain conditions. In the present study, we examined muscle fiber types and enzymatic markers of metabolism for their impact on cycling ME both before and after the HERITAGE endurance exercise‐training program.

In summary, this study allowed us to test the following hypotheses: (1) Cycling endurance training promotes an increase in ME in sedentary individuals; (2) the associations between ME and body composition or glycemic profile are maintained post training when all participants are familiar with the pedaling skill; (3) there is a relationship between the skeletal muscle profile and ME.

## 2 METHODS

The study is based on secondary analysis of data collected in the HERITAGE (HEalth, RIsk factors, Training And GEnetics) Family Study. As previously described (Bouchard et al., 1995; Sarzynski et al., 2022), the main objective of HERITAGE was to investigate the magnitude of the individual differences in response to a standardized endurance exercise program, the importance of familial aggregation, and the genetic profiles associated with the response levels of cardiorespiratory fitness and cardiovascular disease and diabetes risk factors. The present study included a total of 836 participants (*N* = 371 males; *N* = 465 females) from 227 families for whom data on ME were available. Individuals from both European and African descent were recruited, tested, and exercise trained at four clinical centers: Arizona State University and subsequently Indiana University, Laval University, University of Minnesota, and University of Texas at Austin. These participants were tested before and after a 20‐week standardized training program.

A total of 730 participants (319 males and 411 females) from 212 families were available after exercise training and constitute the sample of the present study. These post‐training numbers include only completers, defined as those having completed at least 57 of the 60 exercise training sessions. Participants were aged 17–65 years, required to be sedentary, and exempt of medical conditions and diseases susceptible to interfere with the ability to exercise on cycling ergometers and the main outcomes of the study (Bouchard et al., 1995; Sarzynski et al., 2022). Each participant provided written consent to be enrolled in the study. The HERITAGE protocol was accepted by the ethics committee of each participating academic institution (Washington University, Arizona State University, and subsequently Indiana University, Laval University, University of Minnesota, and University of Texas at Austin). All research was performed in accordance with the Declaration of Helsinki.

### 2.1 Exercise training program

At each clinical center, the training program was conducted on cycle ergometers (Universal Aerobicycle, Cedar Rapids, IA) interfaced with a Med‐net computer system (Universal Gym Mednet, Cedar Rapids, IA) to monitor and control the power output of the ergometers. This allowed the maintenance of the training‐targeted heart rates. The intensity of exercise was initially fixed at a heart rate associated with 55% of baseline maximal oxygen uptake (VO_2_max) for 30 min/session and was gradually increased to 75% of initial VO_2_max for 50 min/session at the end of Week 14. The intensity and duration of exercise were then maintained at this level for the remaining 6 weeks. Frequency of exercise was maintained as much as possible at three sessions per week. In addition to the computerized monitoring of the exercise prescription, each session was supervised by an exercise specialist to ensure that there was no deviation from the prescribed exercise stimulus. Additional details about the exercise program, including VO_2_max measurement, are presented elsewhere (Skinner et al., 2000).

### 2.2 Mechanical efficiency and physical activity

Three exercise tests were administered both before and after the training program (Sarzynski et al., 2022). The present study is based on the data of the submaximal exercise test performed in relative steady state at 50 watts. The choice of an absolute rather than a relative workload was made to replicate a usual free‐living activity whose physiological demand varies between individuals even at a low intensity level. Subjects exercised for a minimum of 12 min at this workload, and the panel of measurements included pulmonary ventilation, VO_2_, VCO_2_, and respiratory exchange ratio. Mechanical efficiency was calculated as the ratio of workload (50 W)/VO_2_ (L/min). Resting VO_2_ was not incorporated in the master datafile, which prevented us from calculating net mechanical efficiency. In this regard, resting VO_2_ corresponds to about 30% of the exercise VO_2_ at 50 W (Wilmore et al., 1998). Thus, gross ME reflects primarily the metabolic processes underlying the demands of the work output. In support of this assumption, we compared gross ME and net ME in the Quebec Family Study and found that they were highly correlated (*p* < 0.0001) in both males (*r* = 0.86) and females (*r* = 0.91).

### 2.3 Body composition measurements

Briefly, underwater weighing combined with measurements of pulmonary residual volume was used to assess body composition (Behnke & Wilmore, 1974). The measurement was performed in the postabsorptive state to quantify body density and compute fat mass, fat‐free mass, and percent body fat using sex‐ and ethnic‐specific equations (Behnke & Wilmore, 1974). A detailed description of these measurements was previously reported (Wilmore et al., 1999).

### 2.4 Glycemic profile

Fasting plasma glucose and insulin levels were determined at baseline and the day following the last exercise session in most participants (94%), as previously described (Boule et al., 2005). For other subjects, the determination was performed between 24 and 72 h after the last exercise session.

### 2.5 Skeletal muscle biology

Biopsies of the vastus lateralis muscle were obtained before and after the training program in a subsample of 78 participants from 19 families from the Quebec site to determine fiber types, capillarity, and marker enzyme activities (Rico‐Sanz et al., 2003). Their mean age and BMI were 33 years and 25 kg/m^2^, respectively. Fibers were designated as Type 1, Type IIA, and Type IIX; their mean area was determined by averaging the cross‐sectional areas of 20 randomly selected fibers of each type. For the measurements of enzyme activities, tissue was homogenized, and the resulting homogenate was used to measure maximal enzymatic activities, as previously described (Rico‐Sanz et al., 2003). The panel of muscle traits included in the present study includes the percentage of type 1, IIA, and IIX fibers, the number of capillaries per fiber type, and the maximal activities of the following enzymes: creatine kinase (CK), phosphorylase (PHOS), hexokinase (HK), phosphofructokinase (PFK), glyceraldehyde phosphate dehydrogenase (GAPDH), lipoprotein lipase (LPL), carnitine palmitoyl transferase (CPT), hydroxyacyl‐CoA dehydrogenase (HADH), citrate synthase (CS), and cytochrome‐c oxidase (COX). All assays were performed according to previously reported methodology (Simoneau et al., 1985, 1986; Simoneau & Bouchard, 1995).

### 2.6 Statistical analyses

Pearson correlations were computed to evaluate the relationship between ME, body composition, and fasting plasma glucose and insulin levels. Associations between ME and body composition and glycemic profile were assessed by comparing participants classified in sex‐specific tertiles (low, middle, and high) of ME using general mixed linear models in the MIXED procedure of SAS (SAS University Edition, version 2022). The nonindependence of family members was considered by modeling the covariance parameters for individuals coming from the same family. Analyses were performed separately in males and females on age‐adjusted data. For body composition variables, analyses were repeated with further adjustment for cardiorespiratory fitness (VO_2_max), while for fasting glucose and plasma insulin, analyses were repeated with further adjustment for cardiorespiratory fitness and percent body fat. For muscle characteristics performed in the subsample of 78 subjects, the same analytical strategy was used. Muscle traits were compared among participants classified into sex‐specific tertiles of ME, both at baseline and after exercise training, after adjustment for age and sex. Analyses were repeated with further adjustment for cardiorespiratory fitness and both cardiorespiratory fitness and percent body fat. Finally, we used a two‐way ANOVA to assess the effect of training and sex on ME on age‐adjusted data in the whole HERITAGE sample. We used the Bonferroni correction to adjust the significance level for multiple testing. For analyses comparing body composition and glycemic profiles (5 variables) before and after exercise training in males and females classified based on sex‐specific tertiles of ME, the adjusted significance level was fixed at *p* = 0.0025 (0.05/20 tests). For analyses of muscle traits (16 traits) before and after exercise training, the adjusted significance level was fixed at *p* = 0.0016 (0.05/32 tests).

## 3 RESULTS

**FIGURE 1:**
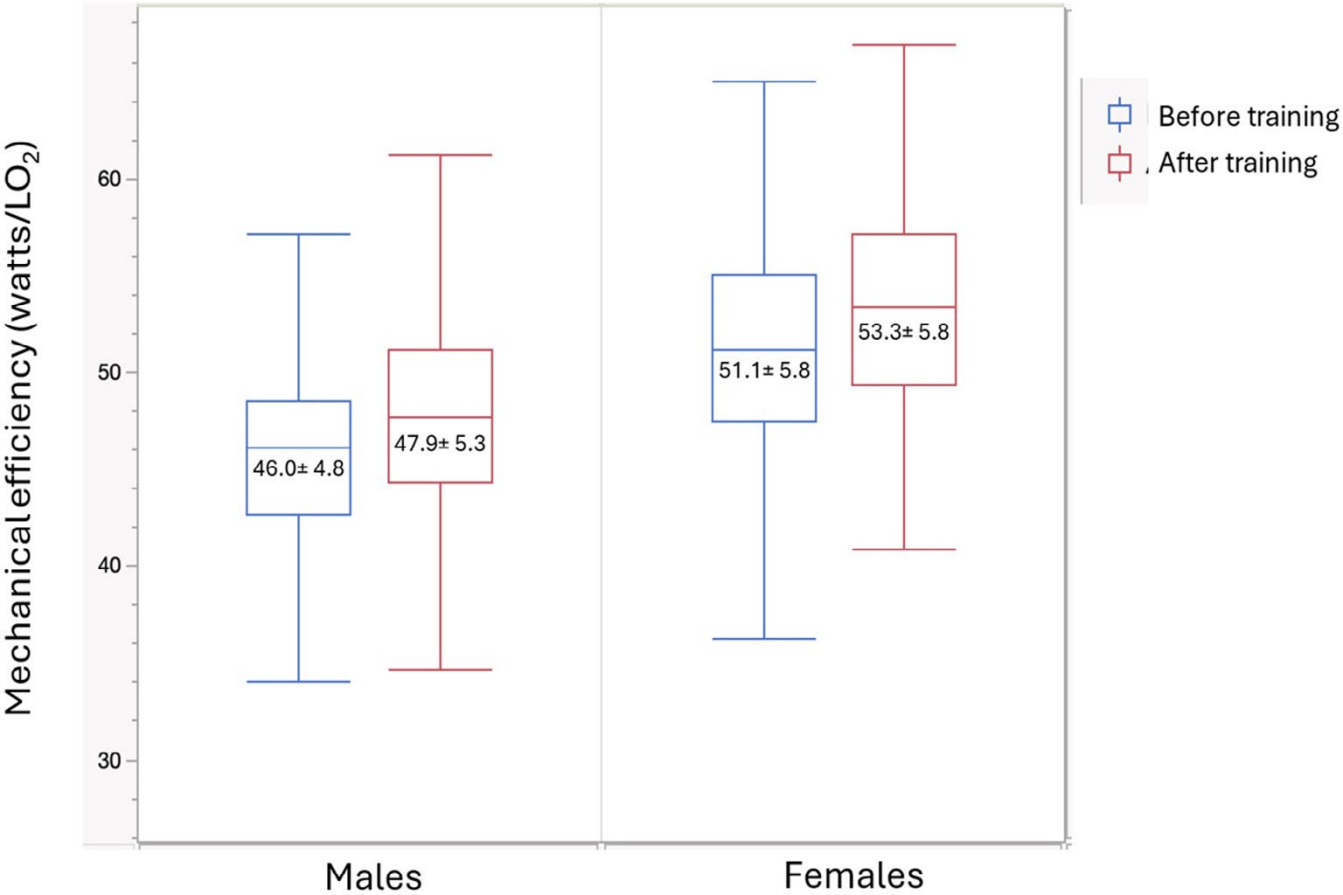
Mechanical efficiency before and after training. The figure presents Whisker plots of mechanical efficiency in males and females before and after training. The box represents the interquartile range and the line inside the median value. Values are means ± SD. Significant effect of training (*p* = 0.0005) and sex (*p* < 0.0001) on age‐adjusted data. No training × sex interaction (*p* > 0.05).

Descriptive characteristics of participants are presented in Table 1. As expected, fat mass and percent body fat were lower in males than in females. ME was significantly greater in females than in males at baseline and following the training intervention. The effect of the training intervention on ME is illustrated in Figure 1. As expected from the experience of elite athletes, the cycling program induced a significant increase in ME, which ranged between 4% and 5% of baseline values and did not differ between sexes.

**TABLE 1:** Descriptive characteristics of participants.

Variables	Before exercise training		After exercise training	
	Males	Females	Males	Females
	(*n* = 371)	(*n* = 465)	(*n* = 319)	(*n* = 411)
Age (years)	35.0 ± 14.1	34.2 ± 12.9		
Mechanical efficiency (watts/L O_2_)	45.8 ± 4.9	51.2 ± 5.8*	47.9 ± 5.3	53.3 ± 5.7*
Body weight (kg)	84.6 ± 17.0	70.4 ± 15.9*	84.1 ± 16.5	69.8 ± 15.0*
VO_2_ max (mL)	2946 ± 569	1843 ± 368*	3388 ± 636	2183 ± 397*
Body mass index (kg/m^2^)	26.9 ± 5.1	26.4 ± 5.9	26.6 ± 4.9	26.3 ± 5.6
Fat mass (kg)	20.2 ± 11.1	23.7 ± 12.0*	19.7 ± 10.8	22.9 ± 11.5*
Percent body fat	22.7 ± 8.8	32.3 ± 9.8*	22.2 ± 8.6	31.5 ± 9.8*
Fasting glucose (mmol/L)	5.21 ± 0.61	4.97 ± 0.66*	5.25 ± 0.58	5.04 ± 0.66*
Fasting insulin (mmol/L)	74.1 ± 58.0	66.8 ± 52.4	68.8 ± 59.8	61.0 ± 50.4

*Note*: Values are means ± SD.

* *p* < 0.01 for differences between males and females.

Table 2 presents correlations between ME and body composition or glycemic profile in male and female participants. At baseline, significant negative correlations were observed in each sex. Higher ME was associated with lower values of body fat and fasting plasma glucose and insulin. The magnitude and statistical significance of these associations were essentially unchanged post intervention when all participants were more homogeneous in terms of their familiarity with cycle exercise.

**TABLE 2:** Correlation coefficients between mechanical efficiency (watts/LO_2_) and body composition or glycemic profile.^a^

Variables	Before exercise training		After exercise training	
	Males	Females	Males	Females
Body weight (kg)	−0.66	−0.65	−0.69	−0.68
Body mass index (kg/m^2^)	−0.60	−0.61	−0.64	−0.65
Fat mass (kg)	−0.54	−0.62	−0.56	−0.63
Percent body fat	−0.40	−0.54	−0.43	−0.56
Fasting glucose (mmol/L)	−0.26	−0.28	−0.23	−0.33
Fasting insulin (mmol/L)	−0.35	−0.37	−0.26	−0.27

^a^ *p* < 0.0001 for all correlations.

The body composition and glycemic profiles of participants classified by tertiles of ME at baseline and after exercise training are presented in Tables 3 and 4, respectively. In both males and females, baseline ME was approximately 25% greater in the upper than in the lower tertile (Table 3). Higher ME was associated with a significantly (*p* < 0.0001) lower age‐adjusted body fatness and plasma fasting glucose and insulin levels in both males and females. These between‐tertile differences remained significant after further adjustment for cardiorespiratory fitness. However, when fasting glucose and insulin levels were further adjusted for both cardiorespiratory fitness and percent body fat, the between‐tertile differences were no longer significant, except for fasting glucose in females (*p* = 0.002). The results following exercise training are presented in Table 4. In both males and females, ME after exercise training was about 25% higher in the upper compared to the lower tertile. As observed at baseline, a higher ME was associated with a more favorable body composition and glycemic profile in both males and females, except for fasting glucose in males where the nominal *p* value (*p* = 0.014) did not reach the Bonferroni adjusted significance level. The associations of ME with body composition and glycemic profile after training were independent of cardiorespiratory fitness as between‐tertile differences remained significant after further adjustment of data for VO_2_ max. No differences between tertiles were observed after further adjustment of fasting glucose and insulin levels for cardiorespiratory fitness and percent body fat.

**TABLE 3:** Association of body composition and glycemic profile with mechanical efficiency (ME) before exercise training.^a^

Variables	Males				Females			
	Low ME	Med ME	High ME	*p* Value	Low ME	Med ME	High ME	*p* Value
	(*N* = 123)	(*N* = 124)	(*N* = 124)		(*N* = 154)	(*N* = 156)	(*N* = 155)	
ME (watts/LO_2_)	40.6 ± 0.2	45.8 ± 0.2	51.1 ± 0.2	<0.000	45.2 ± 0.3	51.3 ± 0.2	56.9 ± 0.3	<0.0001
Body mass index (kg/m^2^)^b^	30.6 ± 0.4	26.5 ± 0.4	24.0 ± 0.4	<0.0001	30.8 ± 0.4	25.6 ± 0.4	23.3 ± 0.4	<0.0001
Fat mass (kg)^b^	28.1 ± 0.9	19.3 ± 0.9	14.9 ± 0.9	<0.0001	32.5 ± 0.9	22.3 ± 0.9	17.5 ± 0.9	<0.0001
Percent body fat^b^	27.5 ± 0.7	22.3 ± 0.7	19.5 ± 0.7	<0.0001	38.1 ± 0.7	31.9 ± 0.7	28.1 ± 0.7	<0.0001
Fasting glucose (mmol/L)^b^, ^c^	5.43 ± 0.05	5.16 ± 0.05	5.06 ± 0.05	<0.0001	5.21 ± 0.06	4.91 ± 0.05	4.83 ± 0.06	<0.0001
Fasting insulin (mmol/L)^b^, ^d^	105.0 ± 5.6	68.9 ± 5.3	56.8 ± 5.4	<0.0001	90.9 ± 4.4	60.2 ± 4.3	51.2 ± 4.5	<0.0001

^a^ Values are age‐adjusted LS means ± SEM in subjects classified based on sex‐specific tertiles of mechanical efficiency (ME). The *N* provided in parentheses is for independent participants.

^b^ After further adjustment for VO_2_ max, differences remained significant (*p* < 0.0001) in both males and females.

^c^ After further adjustment for VO_2_ max and percent body fat, differences remained significant in females (*p* = 0.002), but not in males (*p* = 0.009).

^d^ After further adjustment for VO_2_ max and percent body fat, differences were no longer significant.

**TABLE 4:** Association of body composition and glycemic profile with mechanical efficiency (ME) after exercise training.^a^

Variables	Males				Females			
	Low ME	Middle ME	High ME	*p* Value	Low ME	Middle ME	High ME	*p* Value
	(*N* = 106)	(*N* = 107)	(*N* = 106)		(*N* = 137)	(*N* = 137)	(*N* = 137)	
ME (watts/LO_2_)	42.3 ± 0.3	47.7 ± 0.3	53.7 ± 0.3	<0.0001	47.4 ± 0.3	53.4 ± 0.3	59.0 ± 0.3	<0.0001
Body mass index (kg/m^2^)^b^	30.1 ± 0.4	26.2 ± 0.4	23.8 ± 0.4	<0.0001	30.6 ± 0.4	25.8 ± 0.4	22.6 ± 0.4	<0.0001
Fat mass (kg)^b^	26.4 ± 0.9	18.8 ± 0.9	14.1 ± 0.9	<0.0001	31.6 ± 0.9	22.3 ± 0.9	16.3 ± 0.9	<0.0001
Percent body fat^b^	26.2 ± 0.7	22.0 ± 0.7	18.7 ± 0.7	<0.0001	37.4 ± 0.7	31.7 ± 0.7	26.7 ± 0.7	<0.0001
Fasting glucose (mmol/L)^b^, ^c^	5.38 ± 0.05	5.24 ± 0.05	5.15 ± 0.06	0.014	5.26 ± 0.06	5.03 ± 0.06	4.85 ± 0.06	<0.0001
Fasting insulin (mmol/L)^b^, ^c^	87.6 ± 6.1	65.5 ± 6.1	58.0 ± 6.3	0.001	72.9 ± 4.0	58.8 ± 3.9	47.6 ± 4.0	<0.0001

^a^ Values are age‐adjusted LS means ± SEM in subjects classified based on sex‐specific tertiles of mechanical efficiency (ME). The N provided in parentheses is for experimental replicates, that is, repeated measurements at a different time point.

^b^ After further adjustment for VO_2_ max, differences remained significant (*p* ≤ 0.0025) in both males and females, except in males for fasting glucose (*p* = 0.03).

^c^ After further adjustment for VO_2_ max and percent body fat, value differences are no longer significant.

Results of the substudy on variations in muscle morphology and biology by tertiles of ME at baseline and after exercise training are shown in Table 5. There were no significant differences between ME tertiles for any of the muscle traits either at baseline or after exercise training. Results remained nonsignificant when analyses were repeated using the same ME tertiles as those computed in the whole HERITAGE sample (data not shown).

**TABLE 5:** Muscle characteristics of HERITAGE subjects classified in tertiles of mechanical efficiency^a^.

Variables	Baseline			After exercise training		
	Low ME	Middle ME	High ME	Low ME	Middle ME	High ME
	*N* = 25	*N* = 27	*N* = 26	*N* = 25	*N* = 27	*N* = 25
ME (watts/L O_2_)	46.8 ± 0.4	50.7 ± 0.4	55.6 ± 0.4	48.9 ± 0.4	53.3 ± 0.4	57.8 ± 0.4
% body fat^b^	28.6 ± 1.5	22.1 ± 1.4	21.1 ± 1.5	29.0 ± 1.4	20.6 ± 1.3	21.0 ± 1.4
% type I	44.6 ± 2.6	45.2 ± 2.5	40.0 ± 2.7	46.8 ± 2.9	46.8 ± 2.8	46.2 ± 3.0
% type IIA	36.7 ± 1.7	34.8 ± 1.7	39.9 ± 1.6	36.4 ± 2.3	38.3 ± 2.2	39.8 ± 2.4
% type IIX	18.7 ± 2.4	20.9 ± 2.4	20.3 ± 2.4	16.9 ± 2.6	15.0 ± 2.6	14.2 ± 2.7
Type 1 caps (n/fiber)	4.73 ± 0.16	4.44 ± 0.15	4.59 ± 0.16	5.48 ± 0.21	5.49 ± 0.19	5.29 ± 0.21
Type 2A caps (n/fiber)	4.51 ± 0.16	4.24 ± 0.16	4.47 ± 0.17	5.30 ± 0.19	5.24 ± 0.17	4.94 ± 0.20
Type 2B caps (n/fiber)	3.61 ± 0.15	3.39 ± 0.14	3.42 ± 0.14	4.09 ± 0.18	4.29 ± 0.16	3.81 ± 0.17
CK	399.2 ± 14.7	392.9 ± 14.4	397.6 ± 14.9	424.1 ± 14.7	410.3 ± 16.7	402.8 ± 17.4
PHOS	19.9 ± 1.0	20.1 ± 1.0	21.3 ± 1.0	21.9 ± 1.1	20.9 ± 1.0	21.2 ± 1.1
HK	2.67 ± 0.12	2.67 ± 0.11	2.63 ± 0.11	2.91 ± 0.12	2.81 ± 0.11	2.92 ± 0.12
PFK	58.5 ± 2.6	59.0 ± 2.5	58.7 ± 2.6	63.7 ± 2.7	60.1 ± 2.5	59.9 ± 2.7
GAPDH	435.7 ± 17.5	420.6 ± 17.0	413.7 ± 17.8	456.9 ± 16.8	426.1 ± 16.0	407.5 ± 16.8
LPL	0.88 ± 0.06	0.92 ± 0.06	0.79 ± 0.06	0.88 ± 0.05	0.82 ± 0.05	0.86 ± 0.05
CPT	0.12 ± 0.01	0.13 ± 0.01	0.13 ± 0.01	0.15 ± 0.01	0.15 ± 0.01	0.15 ± 0.01
HADH	16.5 ± 0.8	17.4 ± 0.8	16.9 ± 0.8	20.2 ± 1.0	19.5 ± 1.0	19.7 ± 1.0
CS	11.6 ± 0.8	13.5 ± 0.7	12.7 ± 0.8	15.8 ± 0.9	16.0 ± 0.9	16.3 ± 0.9
COX	7.6 ± 0.5	8.2 ± 0.5	7.4 ± 0.5	8.9 ± 0.5	9.8 ± 0.5	9.2 ± 0.5

*Note*: For all muscle traits, differences between tertiles were not significant (*p* > 0.0016 after Bonferroni correction). Nominal differences were observed for CS in baseline (*p* = 0.02) and after further adjustment for VO_2_ max (*p* = 0.003) and for GAPDH post training (*p* = 0.02) and after further adjustment for VO_2_ max (*p* = 0.04). After further adjustment for VO_2_ max and % body fat, there were no significant differences.

^a^ Values are age‐ and sex‐adjusted means ± SEM. The Vmax of enzymes was expressed in micromoles of substrate per gram of wet weight tissue per minute. Total muscle LPL was expressed in nanomicromoles of FFA transformed per gram of wet weight tissue per minute.

^b^ Significant differences in baseline and after training in age‐ and sex‐adjusted data and after further adjustment for CRF.

## 4 DISCUSSION

The present study aimed to investigate the effects of endurance training on cycling ME in untrained individuals and to explore the associations between ME and body composition and plasma fasting glucose and insulin levels. We undertook a secondary analysis of relevant data from the HERITAGE Family Study, which included a standardized 20‐week cycle endurance exercise program (Bouchard et al., 1995; Sarzynski et al., 2022). As the effects of this intervention on body fat (Wilmore et al., 1999), plasma glucose and insulin (Boule et al., 2005), and muscle biology (Rico‐Sanz et al., 2003; Sarzynski et al., 2022) have been previously reported, the focus here was only on the associations between these variables and cycling ME.

Optimizing ME is important for elite athletes, as the ability to produce as much work as possible for a given oxygen consumption is a potential determinant of performance in sports with high aerobic demands. This is clearly not of paramount importance for sedentary individuals, although there are alternative arguments to justify efforts to improve exercise ME in untrained individuals (Marin‐Couture et al., 2024). Specifically, higher levels of ME are associated with a more favorable morphological and glycemic profile in sedentary adult males and females. In the present study, the lower adiposity and plasma glucose and insulin concentrations in participants with a high ME remained statistically significant after adjustment for cardiorespiratory fitness. However, the potential confounding effect of habituation to cycling exercise on ME is not considered in these analyses and in most studies. The HERITAGE study offered the possibility of documenting two relevant issues: (1) evaluate the impact of a standardized endurance exercise training program on ME in healthy untrained individuals; (2) assess the association of cycling ME with body composition and glycemic profile after training when all participants had become more homogenously familiar with cycle exercise.

The training program induced an increase in cycling ME of 4%–5% of baseline values which corresponds to about 20% of the difference in ME between participants classified in the lower and the upper ME tertiles. The observation is concordant with a prior study (Hopker et al., 2009) that reported an increase of the same order in trained cyclists during the precompetitive season. This new ME level was maintained during the competitive period and then returned to baseline level at the end of the competitive period. These results and those obtained in the present study show that ME is slightly trainable and this adaptation is transient as it disappears when the exposure to the exercise stimulus is reduced, which suggests that the reported training effect on ME is partly explained by a repeated acute effect of exercise on this variable. Interestingly, in one of our previous studies, we observed that human variability in cycling ME in the sedentary state was showing evidence of familial aggregation and a contributing moderate genetic influence (Marin‐Couture et al., 2024).

After 20 weeks of exercise training, when HERITAGE participants had become more familiar with cycling, the associations between ME and the traits considered in the study persisted and remained statistically significant. In addition, comparison of ME tertiles post‐intervention revealed the same level of associations with body composition and glycemic profile, independent of cardiorespiratory fitness. These observations thus extend our previously reported proof of concept study (Marin‐Couture et al., 2024) showing that the associations of ME with body composition and glycemic profile were not solely attributable to variations in habituation to cycle exercise. Our findings also emphasize that the association of cycling ME with the glycemic profile is partly attributable to variation in adiposity, since the statistical adjustment for body composition eliminated statistical significance for most between ME tertile comparisons.

There was an opportunity to investigate some of the potential biological determinants of ME using the substudy of 78 participants in whom muscle biopsies were performed before and after the exercise training program. No associations between cycling ME and body composition or glycemic profile with vastus lateralis muscle fiber type, capillarity, or enzymatic activities were found. The comparison of ME tertiles, which reproduced the differences in ME observed in the whole HERITAGE sample, confirmed the lack of association, as most muscle biology indicators did not differ between tertiles. Therefore, our data could not confirm previously reported results showing that muscle morphology (Horowitz et al., 1994; Layec et al., 2011; Pleguezuelos et al., 2021; Umberger et al., 2006) or enzymatic activities (Goldsmith et al., 2010) are related to ME. However, we acknowledge that the muscle substudy represents a small fraction of the overall HERITAGE cohort and thus firm conclusions cannot be drawn.

Our recent report based on the Quebec Family Study (QFS) showed that females exhibit a higher cycling ME, which is associated with a more favorable glycemic profile despite their higher adiposity (Marin‐Couture et al., 2024). We reproduced these results in the present study both before and after training, with ME in females exceeding the level of males by 11%–12%, together with lower fasting glycemia and higher adiposity. These results suggest that ME could be a mediator of a more favorable metabolic profile in females. Notably, whereas glycemia is lower in the presence of higher ME for both sexes, the same favorable glycemic profile is seen within each ME tertile in females compared to males. Globally, these observations reinforce the notion that ME may have value as a clinical indicator of cardiometabolic health (Franssen et al., 2021; Jabbour et al., 2013; Jabbour & Iancu, 2015; Marin‐Couture et al., 2024). Results of the QFS also suggest that sex differences in ME may be attributable to variations in substrate oxidation (Marin‐Couture et al., 2024). Specifically, the respiratory exchange ratio was found to be significantly greater in females than in males during submaximal exercise. Since the ATP formed/VO_2_ ratio is slightly greater for carbohydrate than for lipid oxidation, this may partly explain the higher level of ME observed in females. However, as previously discussed, the difficulty to standardize the nutritional context of ME measurement may represent a limitation in the study of this issue (Marin‐Couture et al., 2024).

The study has some methodological characteristics deserving attention. The study was based on adults who were confirmed sedentary at baseline; the sample size was rather large for such studies, the endurance exercise program was standardized and fully monitored, only subjects defined as completers (≥95% compliance with the exercise program requirements) were included in the study, and the panel of metabolic traits measured on all participants was comprehensive. On the other hand, the fact that cycling ME was measured as gross ME due to the lack of resting VO_2_ data before the exercise test may be perceived as a limitation of the present study. However, our previous report based on data from the Quebec Family Study using both gross and net cycling ME (Marin‐Couture et al., 2024) suggests that the gross ME measurements in HERITAGE were appropriate to document associations with body composition and glycemic profile, as confirmed by the correlations presented above.

## 5 CONCLUSION

In summary, cycling ME was increased by a cycling endurance exercise training program to the same extent in both sexes. The results confirm that the associations between ME and body composition or glycemic profile are independent of cardiorespiratory fitness. These associations were essentially maintained post‐training when all participants had become familiar with the pedaling skill, suggesting that the ME‐body composition‐glycemic profile relationship is not mainly explained by variations in cycling skill. Adjustment of data for percent body fat suggests that the associations between cycling ME and glycemic profile are at least partly mediated by the level of adiposity. On the other hand, no evidence of associations between muscle fiber types, density of capillaries, and enzymatic activities associated with energy production and ME could be found. We conclude that variations of ME with and between sexes support the hypothesis that exercise ME potentially represents a new predictor of the glycemic profile in healthy individuals.

## AUTHOR CONTRIBUTIONS

CB and JS: Conceived and designed research. CB, MAS, and JS: Performed experiments. AT and LP: Analyzed data. AT, CB, EMC, PS, DJ, GT, MEM, and LP: Interpreted results of experiments. AT and LP: Prepared figures. AT: Drafted manuscript. AT, CB, MAS, JS, EMC PS, DJ, GT, MEM, and LP: Edited and revised manuscript and approved final version of manuscript.

## FUNDING INFORMATION

Data collection of the HERITAGE Family Study was funded by numerous grants from the National Heart, Lung and Blood Institute (NHLBI) of the USA (HL45670, HL47317, HL47321, HL47323, and HL47327).

## CONFLICT OF INTEREST STATEMENT

The authors have no conflict of interest to disclose about the present study.

## ETHICS STATEMENT

The HERITAGE protocol was accepted by the ethics committee of each participating academic institution. See methods for additional details.

## Data Availability

The data used in this study is available upon reasonable request from Mark A. Sarzynski (sarz@mailbox.sc.edu).
